# Supplementary material for: Co-Occurrence of Beckwith–Wiedemann Syndrome and Early-Onset Colorectal Cancer
Source: Cancers (Basel). 2023 Mar 23;15(7):1944. doi: 10.3390/cancers15071944 (PMC10093120; doi:10.3390/cancers15071944)
Supplement: Supplementary file 1 [file cancers-15-01944-s001.zip › Supplementary files/Table S1.pdf]

Table S1. Primers for pyrosequencing analysis.

DMR	Primers	N° CpGs analysed	Genomic Coordinates (hg19)
***H19/IGF2:*IG - DMR**	Forward: 5′-Biotin/ GTGGTTTTTATGACTGTTTTATTTTTGATGA-3′	5	Chr11: 2,021,880-2,022,088
	Reverse: 5′-ACTTCCCCTTCAATCTCACCA -3′		
	Sequencing: 5′-TACAAAATTAATTATAACTATAAAAT-3′		
***IGF2*:alt-TSS-DMR**	Forward: 5′-TGAGGATGGGTTTTTGTTT-3′	6	Chr11: 2,169,328-2,169,581
	Reverse: 5′- Biotin/ CCTCCATCCACCCAAAATAATAT-3′		
	Sequencing: 5′-GGGGTGGAGGGTGTA-3′		
***KCNQ1OT1*:TSS -DMR**	Forward: 5′-GGAGAGTATTGTTTAGGTTAGGTTGTAT -3′	9	Chr11: 2,720,485-2,720,644
	Reverse: 5′ Biotin/CCTCCCCATCTCTCTAAAAAAATTTAA-3′		
	Sequencing: 5′-GGTTAGGTTGTATTGTTG-3′		
***GNAS-AS1*:TSS-DMR**	Forward: 5'-TAGGTTGTAGTGGGGTTAAAGGA-3'	8	Chr20: 57,426,890-57,427,116
	Reverse: 5'-Biotin/ACCTCAAAATTTCCCAAATCCTACTATTCT-3'		
	Sequencing: 5-GTGGGGTTAAAGGAG-3'		
***MEST*:-alt-TSS-DMR**	Forward: 5'-Biotin/AATAAAGGGGGTTTTGTTTTTTTAAT-3'	8	Chr7: 130,131,089-130,131,334
	Reverse: 5'-AACCCACCACCAAACTAAT-3'		
	Sequencing: 5'-TAACCACTATAACCAAAATTAC-3'		
***NNAT*:TSS-DMR** (Murphy, et al., 2012)	Forward: 5'-TAAATTTGTAGGTTAGGGATTGGG-3'	7	chr20:36149325-36149543
	Reverse: 5'- Biotin/CCAAAAAAAAAAAAAAATAATCCATCTACT-3'		
	Sequencing: 5'- TTGTAGGTTAGGGATTG-3'		
***RB1*:Int2-DMR**	Forward: 5'- Biotin/ TGGGGTTAGGAGGTGAAAGTGG -3'	9	chr13:48892910-48893147
	Reverse: 5'-ACTACCCTACCCACCCCCATATAA -3'		
	Sequencing: 5'-AACAAATCCCTTTCTACA -3'		
***NDN*:TSS-DMR**	Forward: 5’-TGGGGGTTTAGGTTGTAAAGTTAG-3’	6	chr15:23932310-23932451
	Reverse: 5'- Biotin/ CACTTCCTCTCCAAAAATCC -3'		
	Sequencing: 5'- AGATTTTTATTTTGTTTTGATATG -3'		

The NNAT:TSS-DMR primers were previously reported in Murphy, S. K., Z. Huang and C. Hoyo. (2012), 'Differentially Methylated Regions of Imprinted Genes in Prenatal, Perinatal and Postnatal Human Tissues', *PLoS One* Vol. 7, No. 7, pp. e40924.
