# Supplementary material for: Co-Occurrence of Beckwith–Wiedemann Syndrome and Early-Onset Colorectal Cancer
Source: Cancers (Basel). 2023 Mar 23;15(7):1944. doi: 10.3390/cancers15071944 (PMC10093120; doi:10.3390/cancers15071944)
Supplement: Supplementary file 1 [file cancers-15-01944-s001.zip › Supplementary files/Table S2.pdf]

Table S2. Primers for amplification and Sanger sequencing of CFTR variants.

Variant	Primers	Coordinates of the amplified region in hg19
F1052V	Forward:<br>5′- AGGTTGGGCTCAGATCTGTG -3′	chr7:117304740-117304901
	Reverse:<br>5′- GAGCACTGGGTTCATCAAGC -3′	
Q1352E	Forward:<br>5′- TCAAAGAATGGCACCAGTGT -3′	chr7:117251482-117251720
	Reverse:<br>5′- TTCAAAGTAAGGCTGCCGTC -3′	
