# Supplementary material for: Co-Occurrence of Beckwith–Wiedemann Syndrome and Early-Onset Colorectal Cancer
Source: Cancers (Basel). 2023 Mar 23;15(7):1944. doi: 10.3390/cancers15071944 (PMC10093120; doi:10.3390/cancers15071944)
Supplement: Supplementary file 1 [file cancers-15-01944-s001.zip › Supplementary files/Table S3.pdf]

Table S3. Primers for expression analysis.

Gene	Primers
KCNQ1OT1	Forward:5′- AGCCAGACAGAAGCCCAATA-3′
	Reverse:5′- TGGCCTAACATATCATCCCTCC-3′
KCNQ1	Forward:<br>5′- AACACACAGAAGGGGACTGC-3′
	Reverse:<br>5′- GCCTGTGATTCTCCACGTTT-3′
CDKN1C	Forward:<br>5′- AGAGATCAGCGCCTGAGAAG-3′
	Reverse:<br>5′- CACCTTGGGACCAGTGTACC-3′
GAPDH	Forward:<br>5′-TCTCCTCTGACTTCAACAGCGACA-3′
	Reverse:<br>5′- CCCTGTTGCTGTAGCCAAATTCGT-3′
