# Supplementary figures and images for: Co-Occurrence of Beckwith–Wiedemann Syndrome and Early-Onset Colorectal Cancer
Source: Cancers (Basel). 2023 Mar 23;15(7):1944. doi: 10.3390/cancers15071944 (PMC10093120; doi:10.3390/cancers15071944)

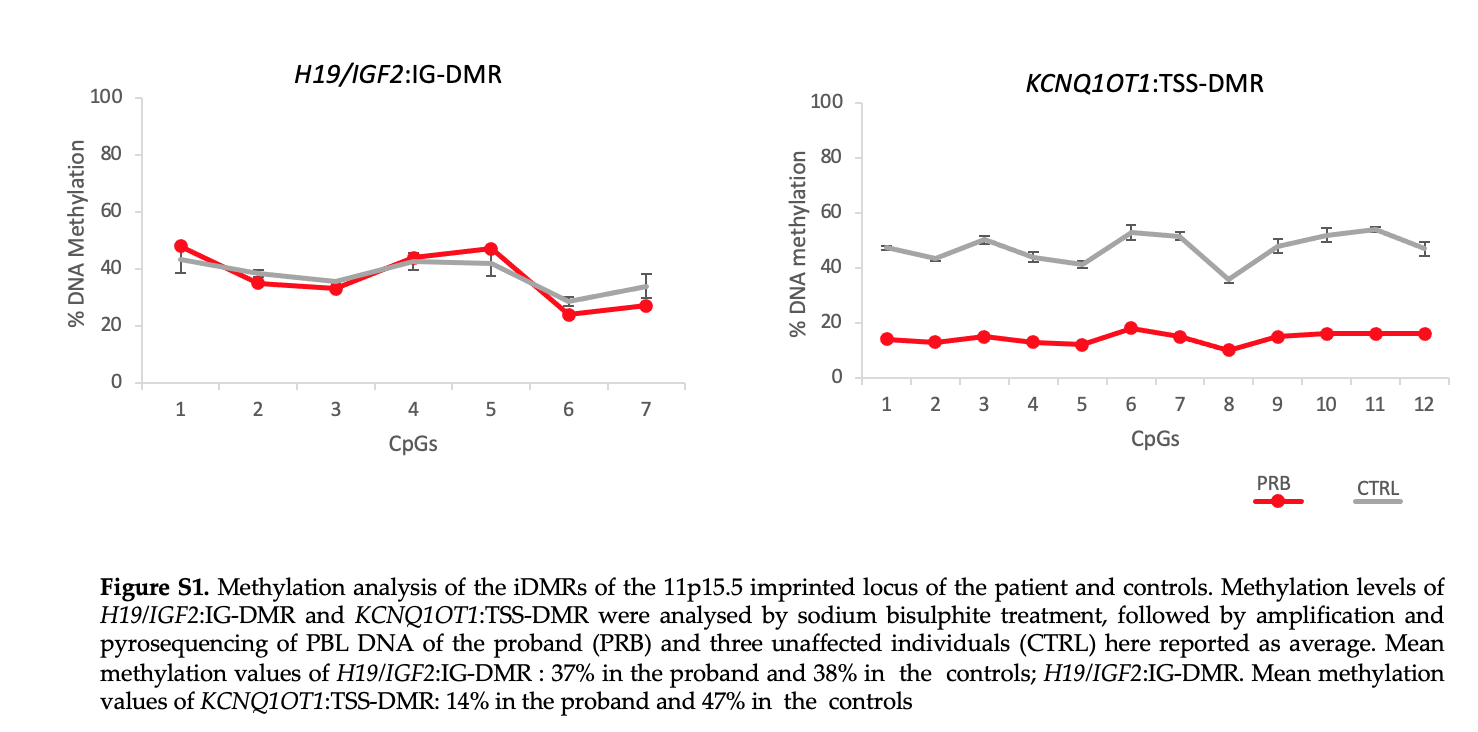

Supplement: Supplementary file 1 [file cancers-15-01944-s001.zip › Supplementary files/Figure S1.png]

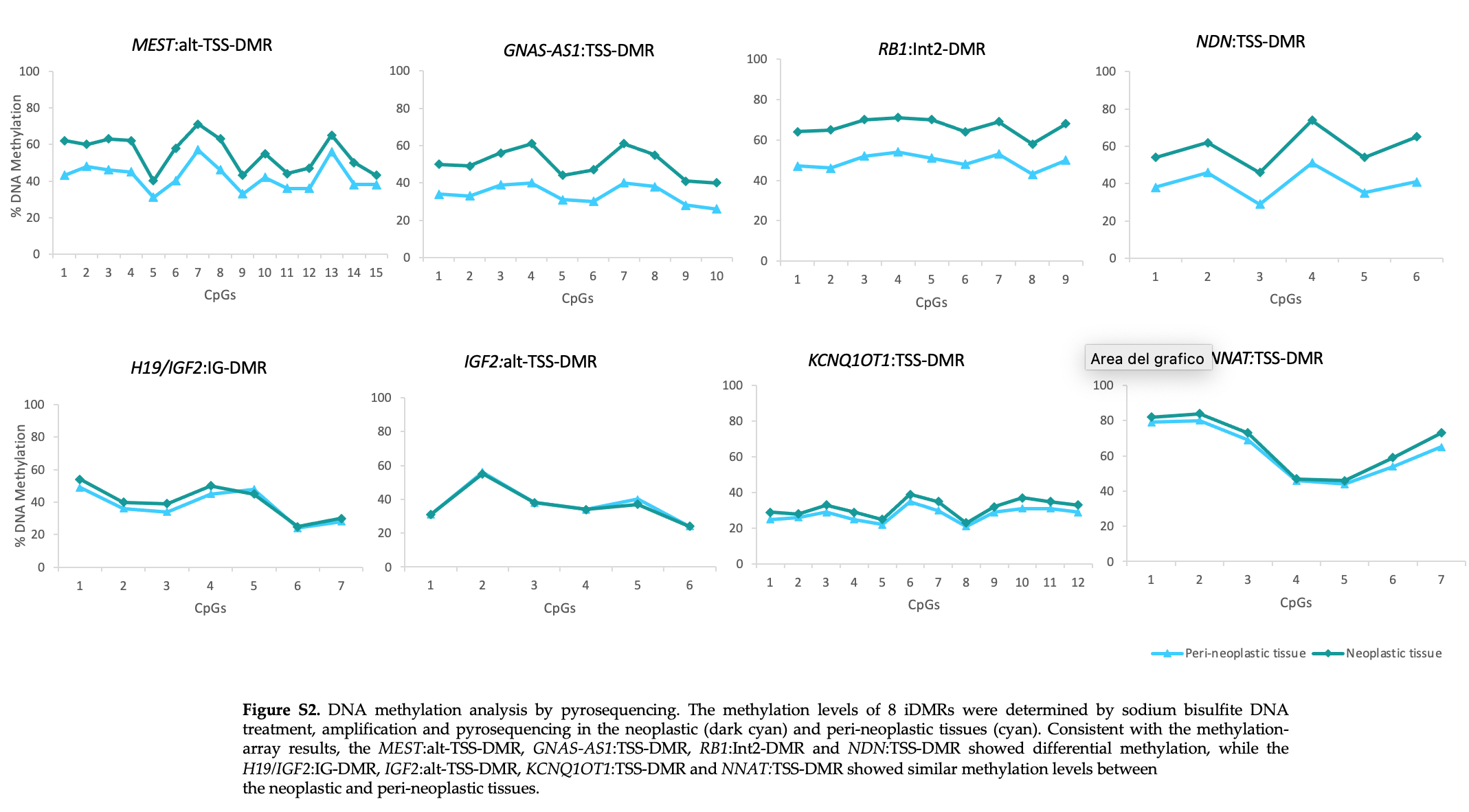

Supplement: Supplementary file 1 [file cancers-15-01944-s001.zip › Supplementary files/Figure S2.png]

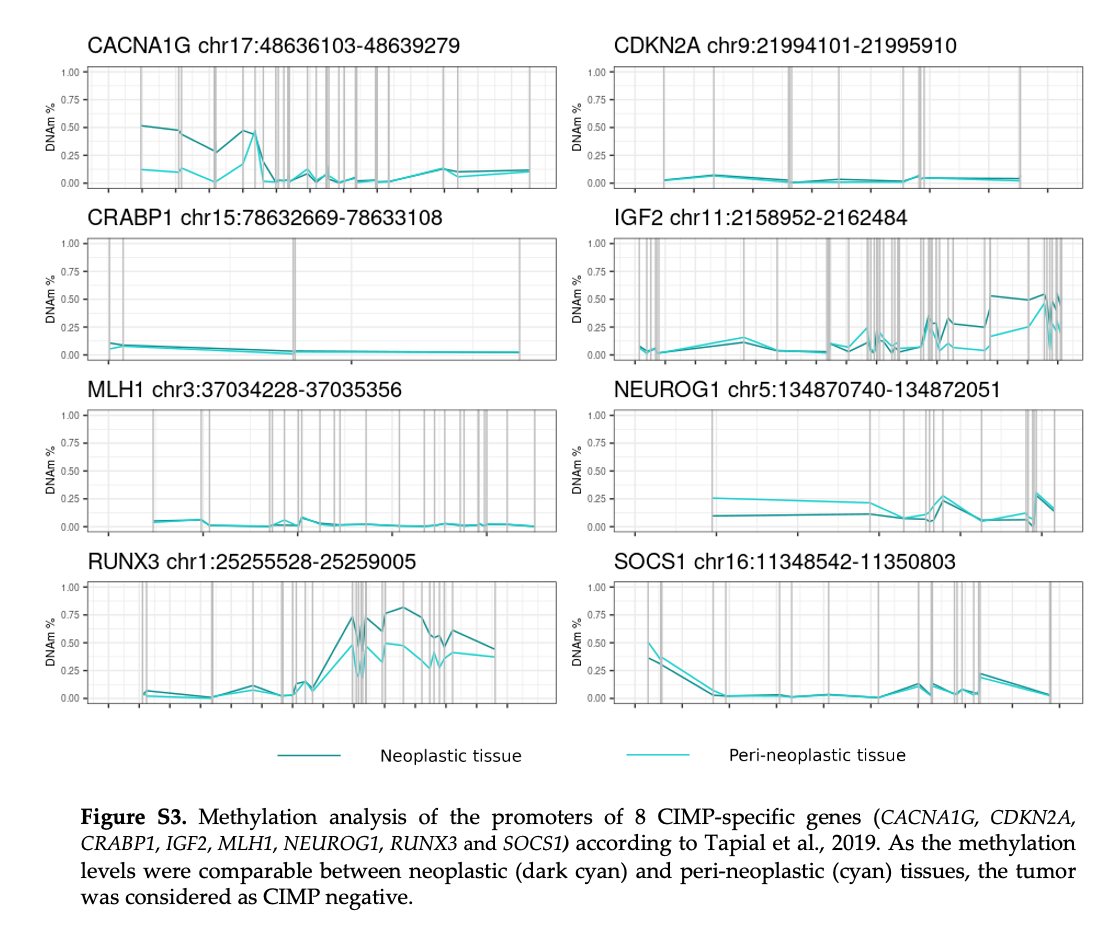

Supplement: Supplementary file 1 [file cancers-15-01944-s001.zip › Supplementary files/Figure S3.png]
